# Mitochondria‐associated endoplasmic reticulum membranes tethering protein VAPB‐PTPIP51 protects against ischemic stroke through inhibiting the activation of autophagy

**DOI:** 10.1111/cns.14707

**Published:** 2024-04-07

**Authors:** Mingyang Li, Yonggang Zhang, Guixiang Yu, Lijuan Gu, Hua Zhu, Shi Feng, Xiaoxing Xiong, Zhihong Jian

**Affiliations:** ^1^ Department of Neurosurgery Renmin Hospital of Wuhan University Wuhan China; ^2^ Department of Ophthalmology Renmin Hospital of Wuhan University Wuhan China; ^3^ Central Laboratory Renmin Hospital of Wuhan University Wuhan China

**Keywords:** autophagy, ischemic stroke, MAMs, PI3K/AKT/mTOR, VAPB‐PTPIP51

## Abstract

**Aims:**

Mitochondria‐associated endoplasmic reticulum membranes (MAMs) serve as a crucial bridge connecting the endoplasmic reticulum (ER) and mitochondria within cells. Vesicle‐associated membrane protein‐associated protein B (VAPB) and protein tyrosine phosphatase interacting protein 51 (PTPIP51) are responsible for the formation and stability of MAMs, which have been implicated in the pathogenesis of various diseases. However, the role of MAMs in ischemic stroke (IS) remains unclear. We aimed to investigate the role of MAMs tethering protein VAPB‐PTPIP51 in experimental cerebral ischemia.

**Methods:**

We simulated cerebral ischemia–reperfusion injury (CIRI) by using a mouse middle cerebral artery occlusion (MCAO) model.

**Results:**

We observed a decrease in VAPB‐PTPIP51 expression in the brain tissue. Our findings suggested compromised MAMs after MCAO, as a decreased mitochondria–ER contact (MERC) coverage and an increased distance were observed through the transmission electron microscope (TEM). Upon VAPB or PTPIP51 knockdown, the damage to MAMs was exacerbated, accompanied by excessive autophagy activation and increased reactive oxygen species (ROS) production, resulting in an enlarged infarct area and exacerbated neurological deficits. Notably, we observed that this damage was concomitant with the inhibition of the PI3K/AKT/mTOR pathway and was successfully mitigated by the treatment with the PI3K activator.

**Conclusions:**

Our findings suggest that the downregulation of VAPB‐PTPIP51 expression after IS mediates structural damage to MAMs. This may exacerbate CIRI by inhibiting the PI3K pathway and activating autophagy, thus providing new therapeutic targets for IS.

## Graphical abstract

The downregulation of VAPB‐PTPIP51 expression after ischemic stroke mediates structural damage to mitochondria‐associated endoplasmic reticulum membranes, which may exacerbate CIRI by regulating the PI3K pathway and activating autophagy.

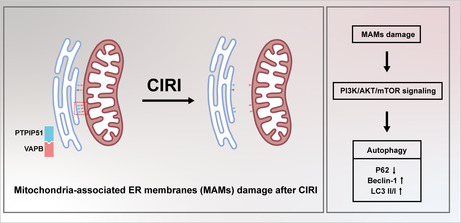

## 1 INTRODUCTION

Ischemic stroke (IS) is the second leading cause of death and the third of combined death and disability, as recently demonstrated by the increasing incidence with age and a trend toward younger individuals.^1^, ^2^ The clinical importance of IS extends beyond high morbidity and disability rates to long‐term sequelae, significantly impacting patients' quality of life and imposing a substantial social burden, especially in developing countries.^3^, ^4^ However, immediate clinical interventions for improving cerebral revascularization or effective pharmacological approaches against IS remain limited, suggesting an urgent need for new therapies.^5^, ^6^

Research suggests that the sudden interruption of cerebral blood flow results in an immediate shortage of oxygen and glucose, triggering disrupted energy supply and consequent pathophysiological processes, including cellular excitotoxicity, mitochondrial dysfunction, neuroinflammation, endoplasmic reticulum (ER) stress, and cell death.^7^ Although mitochondria and the ER have been demonstrated to play pivotal roles in the pathology of IS, they are indispensable organelles that do not function independently, instead, they are tightly interconnected through specialized structures known as mitochondria‐associated endoplasmic reticulum membranes (MAMs).^8^, ^9^, ^10^ MAMs comprise several essential components, including mitofusin 2 (Mfn2),^11^ glucose‐regulated protein 75 (Grp75),^12^ dynamic‐related protein 1 (Drp1),^13^ alpha‐synuclein,^14^ vesicle‐associated membrane protein‐associated protein B (VAPB), and protein tyrosine phosphatase‐interacting protein 51 (PTPIP51),^15^ in addition to a few ER‐resident Chaperones.^16^, ^17^ These components actively participate in protein folding and proton pump activities, thereby contributing to the maintenance of MAMs stability.^18^, ^19^

VAPB and PTPIP51 constitute a set of tethering proteins, which play vital roles in the formation and maintenance of MAMs. VAPB is a membrane protein located in the ER membrane, whereas, PTPIP51 is situated in the outer mitochondrial membrane.^15^, ^20^, ^21^ The interaction between VAPB and PTPIP51 within MAMs is involved in regulating mitochondria–ER contact (MERC), modulating Ca^2+^ transport,^15^ autophagy,^22^, ^23^ phospholipid synthesis,^24^ and neuronal synaptic activity.^25^ MAMs play a crucial role in maintaining cellular physiological homeostasis.^26^, ^27^, ^28^ Disturbances in the structure or function of MAMs have been implicated in the pathogenesis of cancer,^29^ diabetes,^30^ and neurodegenerative disorders, such as Alzheimer's disease^31^ and amyotrophic lateral sclerosis.^32^ Exploring the potential functions of MAMs in diverse diseases has deepened our understanding of the pathophysiology and has provided new directions for future research and therapeutic approaches.^18^, ^31^, ^32^ However, the exact role and mechanism of VAPB‐PTPIP51 on MAMs in the IS remain unreported and require further elucidation.

In this study, we employed an in vivo middle cerebral artery occlusion (MCAO) model to simulate cerebral ischemia–reperfusion injury (CIRI) and investigated the structure changes in MAMs mediated by VAPB‐PTPIP51, exploring their potential involvement in the pathological processes of IS. This study offers new insights that enhance our understanding of IS and provides novel therapeutic strategies aimed at targeting MAMs for the treatment of IS.

## 2 METHODS

### 2.1 Animals

All animal protocols used in our study were approved by the Medical Ethics Committee of Renmin Hospital of Wuhan University. In this study, 300 male C57BL/6J mice (aged 8–10 weeks) were obtained from the Hunan Silaikejingda (SJA) Laboratory Animal. The mice were randomly grouped based on their body weight, with 40 in the sham group and 40 in the vehicle group. Additionally, 60 mice were allocated to each of the scramble, VAPB‐KD, and PTPIP51‐KD groups. Within these three groups, 40 mice were further subdivided into PI3K activator treatment groups and vehicle treatment groups, with 20 mice in each. The mice were acclimated for a week before the commencement of the experiment.

### 2.2 Lentiviral injection

As previously reported, cortical injection of lentiviral vectors (LV) carrying shRNA was performed using a stereotaxic instrument.^33^, ^34^ The shRNA oligonucleotide were 5′‐CCGGCGGAAGACCTTATGGATTCAACTCGAGTTGAATCCATAAGGTCTTCCGTTTTTG‐3′, targeting VAPB, and 5′‐CCGGGAACTGCCAGACGTCACTAATCTCGAGATTAGTGACGTCTGGCAGTTCTTTTTG‐3′ targeting PTPIP51. Briefly, three cortical injections were administered to the subjected mice, positioned 0.3 mm anterior to the bregma, 0.8 mm and 1.9 mm posterior to the bregma; all injection points were located ipsilateral to the infarct area, 3 mm lateral to the sagittal suture, and 2 mm deep. Concentrated LV suspension (2 × 10^9^ TU/mL) was injected. We kept the syringe at target points for 7 min and removed it slowly to ensure optimal lentivirus absorption. A green fluorescent protein‐expressing lentiviral vector was utilized in the scramble group. Mice were subjected to MCAO 5 days after the LV injection.

### 2.3 MCAO animal model and drug administration

The MCAO model has been described in our previously published studies.^33^, ^34^ In brief, mice were anesthetized with isoflurane by mask oxygen administration, and then positioned in a supine posture and placed on a thermostatic heating pad during the entire procedure. We isolated and ligated the common carotid artery (CAA) and external carotid artery, a microarterial clip was used to block the distal end of the CAA. We made a small incision in the CAA, then inserted a monofilament thread into the CAA through the incision and extended to occlude the middle cerebral artery. Then the thread was removed 2 h after the induction of ischemia to restore blood flow. The sham group underwent identical surgical procedures without thread insertion. Additionally, mice in the treatment group received an intraperitoneal injection of PI3K activator 740 Y‐P (MCE, HY‐P0175) at the onset of reperfusion.

### 2.4 Neurological scores

The Longa scale was employed for single‐blind assessment of neurological scores 1 day after reperfusion. Score 0, no neurological deficit; score 1, failure to fully extend the right forepaw; score 2, circling to the right; score 3, falling to the right, and score 4, no spontaneous motor activity, exhibiting a depressed level of consciousness.

### 2.5 Infarction volume measurement

The mice were euthanized with an overdose of isoflurane 1 day after reperfusion. We removed the brain tissue into a brain section mold and sliced it into four consecutive slices. Then, the slices were incubated in a 2% 2,3,5‐triphenyl‐tetrazolium chloride (TTC) solution in the dark for 20 min. After staining, the slices were fixed in 4% paraformaldehyde. The infarct volume was assessed in a blinded manner using ImageJ (Media Cybernetics Inc) by an evaluator, as described in our previous studies.^35^, ^36^

### 2.6 ROS, malondialdehyde, and glutathione measurement

We euthanized the mice 1 day after MCAO, removed the brain tissue rapidly, and sliced it into 4.0 μm sections. Then, the sections were incubated for 30 min at 37°C in dihydroethidium (DHE, Sigma–Aldrich) solution, protected from light. The sections were rinsed thrice after incubation, then we randomly selected three brain slices from the ischemic region and observed ROS‐positive cells in the penumbra with an automated fluorescence microscope (Olympus, Japan). Fluorescence intensity was quantified and averaged using ImageJ, with the sham group serving as a reference for quantification.

Mice were euthanized with an overdose of isoflurane 1 day after reperfusion. Malondialdehyde (MDA) and glutathione (GSH) levels were evaluated in the collected fresh brain tissues using commercial assay kits (EYKITS, Shanghai, China), rigorously conducted following the protocol. Statistical analysis was performed using five mice in each group.

### 2.7 Western blotting

We harvested the total protein from the ischemic penumbra 1 day after ischemia/reperfusion (I/R) using RIPA lysis buffer, then loaded the samples for SDS‐PAGE, then we used the PVDF membranes (Millipore) to transfer proteins. The PVDF membranes were blocked with 5% bovine serum albumin (BSA) solution for 1 h, then incubated overnight at 4°C with the following primary antibodies, anti‐VAPB (95339, Cell Signaling Technology), anti‐PTPIP51 (20641‐1‐AP, Proteintech), anti‐Mfn2 (12186‐1‐AP, Proteintech), anti‐β‐actin (1967, Cell Signaling Technology), anti‐Beclin‐1 (2738, Cell Signaling Technology), anti‐P62 (39749, Cell Signaling Technology), anti‐LC3I/II (12741, Cell Signaling Technology), anti‐PI3K (4257, Cell Signaling Technology), anti‐p‐PI3K (17366, Cell Signaling Technology), anti‐AKT (9272, Cell Signaling Technology), anti‐p‐AKT (3038, Cell Signaling Technology), anti‐mTOR (2972, Cell Signaling Technology) and anti‐p‐mTOR (2971, Cell Signaling Technology). The membranes were rinsed three times with phosphate‐buffered saline (PBS)/0.1% Tween, then incubated with secondary antibodies for 1 h. Images were observed using a Bio‐Rad ChemiDoc western blot analysis system, and protein expression levels were normalized to β‐actin with ImageJ.

### 2.8 Immunofluorescence staining

The brain tissues were collected in each group following perfusion with ice‐cold PBS 1 day after MCAO, and fixed with 4% paraformaldehyde for 48 h, then cut into slices. The slices were incubated in 0.3% Triton X for 10 min and blocked with 5% fetal bovine serum for 60 min at room temperature. The slices were incubated with the following primary antibodies overnight at 4°C after rinsing with PBS thrice, anti‐VAPB (66191, Proteintech), anti‐PTPIP51 (20641, Proteintech), and anti‐NeuN (94403, Cell Signaling Technology). The slices were washed thrice and then incubated with an Alexa 488‐conjuated antibody (ANT024S; Millipore, Billerica, MA) or Alexa 594‐conjugated antibody (ANT029S, Millipore, Billerica, MA) for 2 h at room temperature. DAPI was used to stain cell nuclei after washing with PBS. We randomly selected three brain sections from the ischemic region of each animal and observed VAPB, PTPIP51, NeuN immuno‐positive cells in the penumbra with an automated fluorescence microscope (Olympus, Japan). The number of positively stained cells was calculated and averaged with ImageJ. Statistical analysis was performed using five mice in each group.

### 2.9 Transmission electron microscope

The anesthetized mice brain tissues were collected following perfusion with PBS and cut into sections less than 1 mm thick. The fresh tissue sections were immersed in a fixative for transmission electron microscope (TEM) observation at 4°C for 4 h and subsequently fixed with 1% osmium tetroxide for 120 min. All sections were then dehydrated with gradient alcohol, embedded, and cut into ultrathin sections. Finally, the ultrastructures of MAMs were imaged with a TEM (Xarosa, EMSIS ASIA). Quantification of MERC coverage and distance per cell area was performed using ImageJ as described.^37^ Statistical analysis was performed using five mice in each group.

### 2.10 Statistical analysis

All data are shown as the mean ± SD. GraphPad Prism was used to perform statistical analysis. All data underwent normality testing using both the Shapiro–Wilk and Kolmogorov–Smirnov tests. Subsequently, comparisons between two groups were analyzed using the *t*‐test, while comparisons among multiple groups were assessed via one‐way ANOVA with Tukey's post hoc test. The results were considered statistically significant with a *p* value of <0.05.

## 3 RESULTS

### 3.1 The composition and ultrastructure of MAMs were impaired after CIRI

**FIGURE 1:**
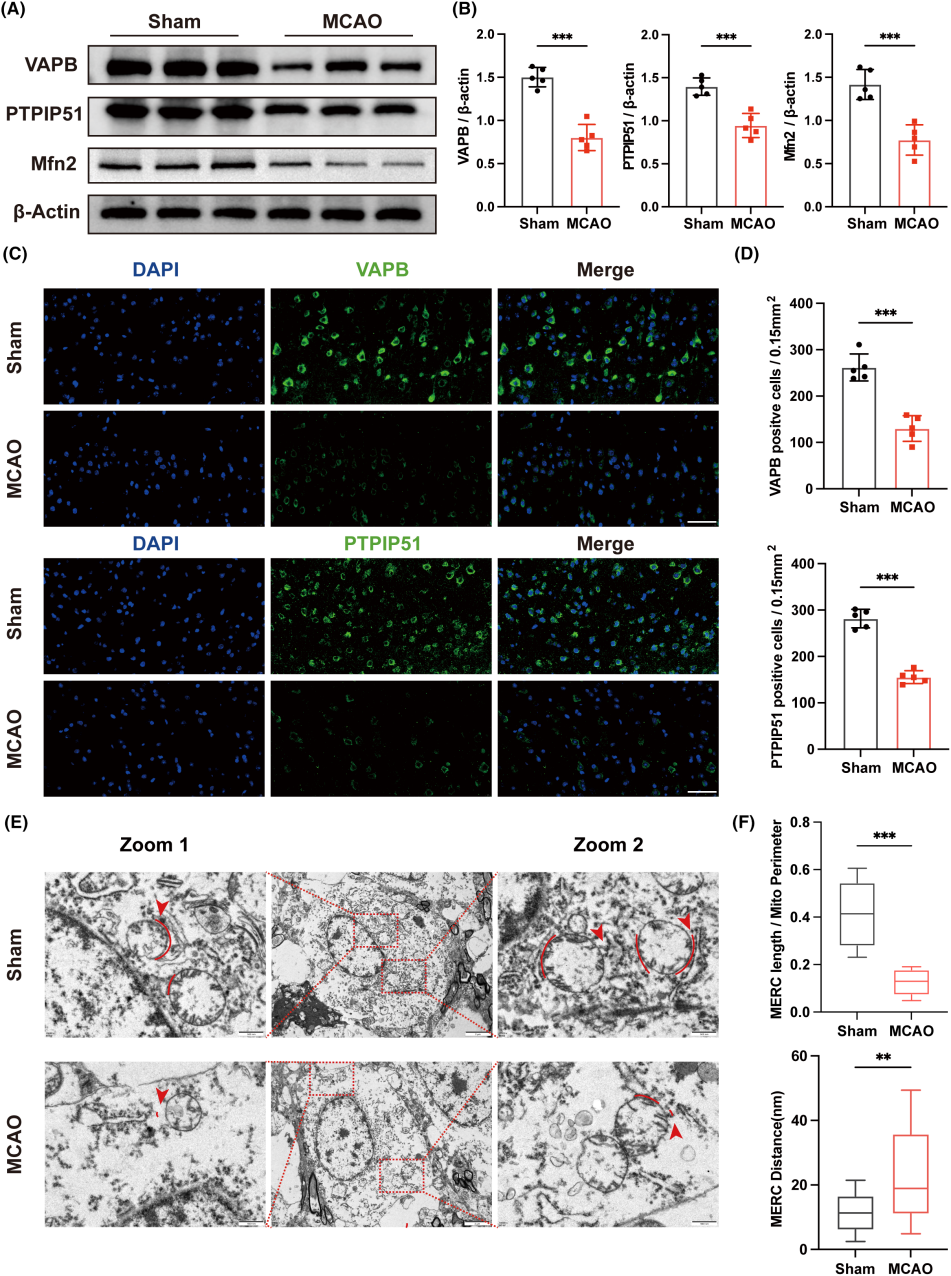
The expression of MAMs tethering proteins and ultrastructure of MAMs in mice 1 day after middle cerebral artery occlusion (MCAO). (A) Western blot bands of the protein expression of Vesicle‐associated membrane protein‐associated protein B (VAPB), PTIPI51, and Mfn2 in mice 1 day after MCAO, *n* = 5. (B) Quantitative analysis of the expression of VAPB, PTPIP51, and Mfn2 detected by Western blot. (C) Immunofluorescence staining of VAPB and PTPIP51 in penumbra region. Scale bar = 50 μm, *n* = 5. (D) Quantitative analysis of VAPB‐positive and PTPIP51‐positive cells stained by immunofluorescence staining. (E) Representative images of mitochondria–ER contact (MERC) after MCAO observed by TEM. Scale bar = 500 nm, *n* = 3/group. (F) Quantitative analysis of MERC parameters, including MERC length/Mito perimeter and MERC distance in TEM images. All values represent mean ± SD, **p < 0.01, ***p < 0.001.

MAMs play a critical role in maintaining cellular physiological homeostasis. VAPB and PTPIP51 serve as anchoring site between the ER and mitochondria, playing a vital role in maintaining the normal structure and physical function of MAMs. To investigate the changes in MAMs in CIRI, we conducted an MCAO model in male C57BL/6J mice. Western blot analysis showed that VAPB and PTPIP51 were downregulated 1 day after MCAO (Figure 1A,B). Besides, compared to that in the sham group, Mfn2, another component of MAMs, was decreased in CIRI mice (Figure 1A,B). Moreover, we performed an immunofluorescence staining to detect the expression of VAPB and PTPIP51 in the penumbra region. The results revealed that the number of VAPB‐positive and PTPIP51‐positive cells were significantly reduced (Figure 1C,D). Furthermore, we conducted TEM to detect the mitochondria–ER interaction after CIRI. As described,^37^ we analyzed the MERC coverage rate of mitochondria and the distance between the ER and mitochondria. The results revealed a reduced MERC coverage rate and an increased distance between the ER and mitochondria in mice following 1 day of CIRI (Figure 1E,F). These results indicated that the MAMs structure was compromised after CIRI.

### 3.2 Knockdown of VAPB‐PTPIP51 exacerbated CIRI in mice

**FIGURE 2:**
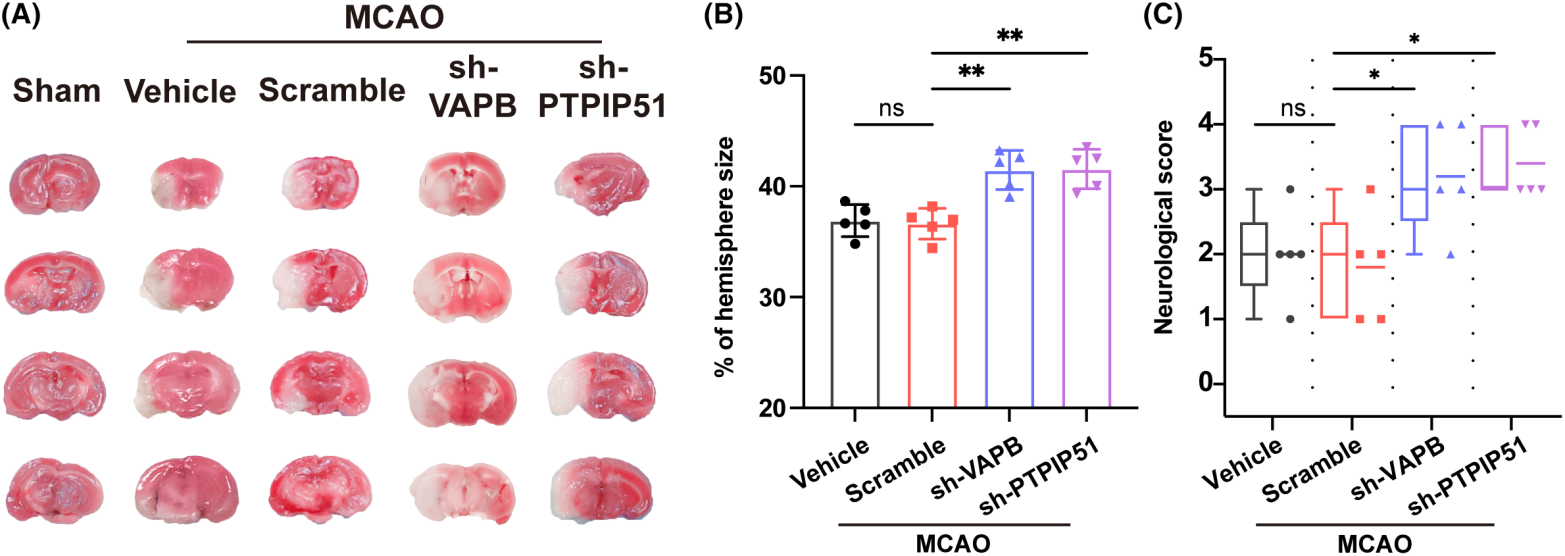
The effect of vesicle‐associated membrane protein‐associated protein B (VAPB)‐PTPIP51 knockdown on mice after MCAO. (A) Representative 2,3,5‐triphenyl‐tetrazolium chloride (TTC) staining of mouse brains 1 day after MCAO. *n* = 5/group. The white color represents the infarcted area, and the red color represents the noninfarcted area. (B) Quantitative analysis of the infarct size using contralateral hemisphere as a reference and presented as a percentage, *n* = 5. (C) Neurological deficits of MCAO mice 1 day after MCAO. *n* = 5. All values represent mean ± SD, ns, nonsignificance, *p < 0.05, **p < 0.01.

The experimental results above have already demonstrated the presence of MAMs damage after stroke. To further elucidate whether MAMs impairment plays a role in the subsequent pathological process of CIRI, we respectively knocked down the expression of the key MAMs tethering protein, VAPB and PTPIP51, by injection of LV‐sh VAPB and LV‐sh PTPIP51 5 days before MCAO. TTC staining showed no significant difference between the vehicle and scramble groups 1 day after MCAO. However, compared with the scramble group, VAPB or PTPIP51 knockdown resulted in an increased infarct volume, respectively (Figure 2A,B). Consistently, the results showed a worse neurological deficit after the VAPB or PTPIP51 knockdown in mice (Figure 2C).

### 3.3 Knockdown of VAPB‐PTPIP51 exacerbated the damage to MAMs after CIRI

**FIGURE 3:**
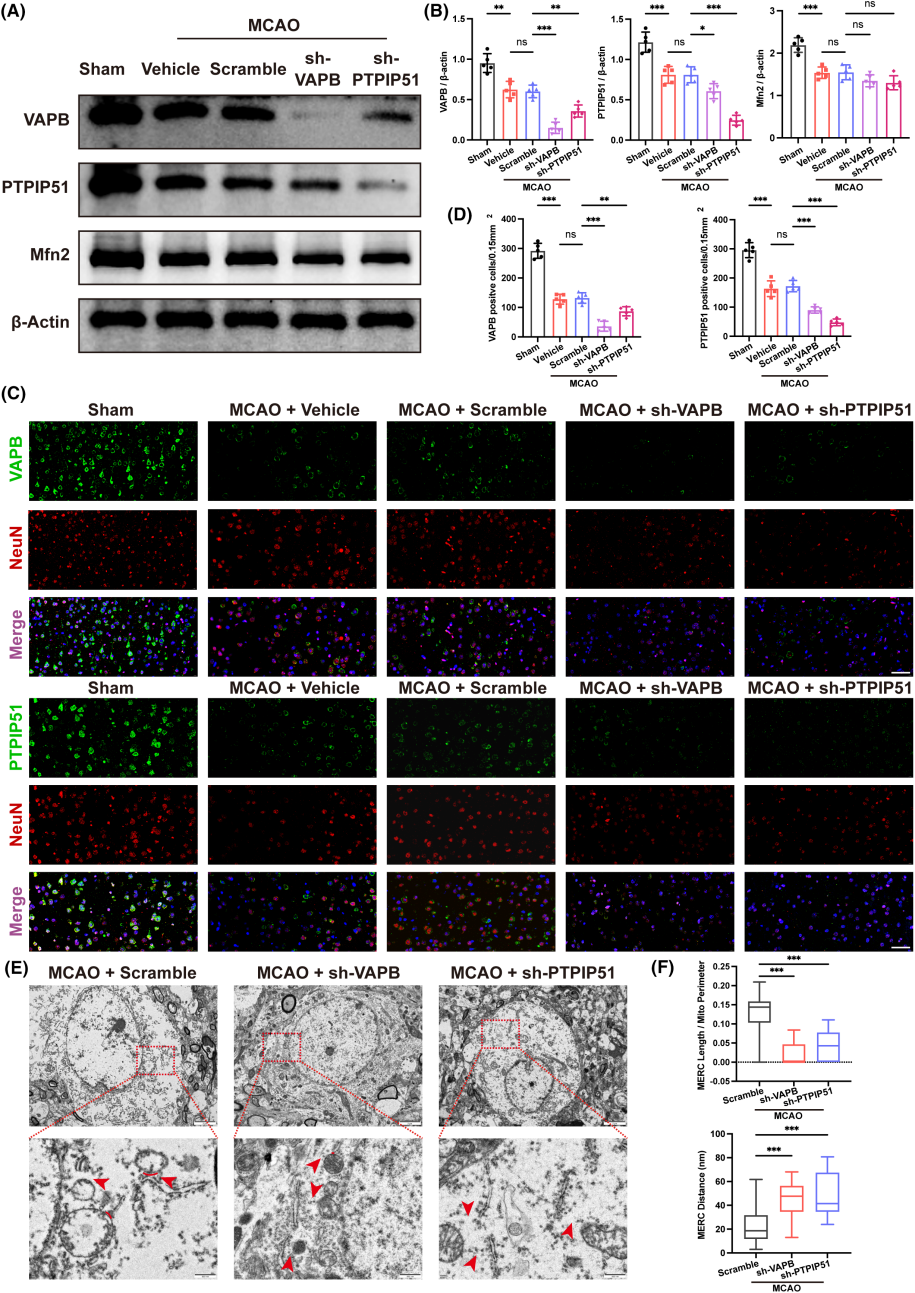
The effect of vesicle‐associated membrane protein‐associated protein B (VAPB)‐PTPIP51 knockdown on MAMs in mice after MCAO. (A) Representative Western blot bands of MAMs tethering proteins in VAPB or PTPIP51 knockdown mice 1 day after MCAO. *n* = 5. (B) Quantitative analysis of the expression of VAPB, PTPIP51, and Mfn2 detected by Western blot. (C) Representative immunofluorescence staining of VAPB, PTPIP51, and NeuN in the penumbra area after LV injection. Scale bar = 50 μm, *n* = 5. (D) Quantitative analysis of VAPB or PTPIP51‐positive cells stained by immunofluorescence staining. (E) Representative images to show the damaged mitochondria–ER contact (MERC) after VAPB or PTPIP51 knockdown observed by TEM. Scale bar = 500 nm, *n* = 3/group. (F) Quantitative analysis of MERC length/Mito perimeter and MERC distance in TEM images. All values represent mean ± SD, ns, nonsignificance, *p < 0.05, **p < 0.01, ***p < 0.001.

Western blot analysis showed that there was no obvious difference between the vehicle and the scramble groups in the expression of VAPB, PTPIP51, and Mfn2. However, VAPB knockdown reduced the PTPIP51. Meanwhile, PTPIP51 knockdown also downregulated the expression of VAPB (Figure 3A,B). Besides, immunofluorescence staining showed consistent results, indicating that VAPB knockdown decreased the number of VAPB‐positive and PTPIP51‐positive cells, with the same effect observed in the PTPIP51 knockdown group. Notably, VAPB or PTPIP51 knockdown reduced the number of NeuN‐positive cells in the penumbra area compared with that in the scramble group (Figure 3C,D). Furthermore, we observed the changes in MERC and observed that compared to that in the scramble group, VAPB or PTPIP51 knockdown reduced the MERC coverage rate and increased the distance between the ER and mitochondria, respectively (Figure 3E,F). These results indicated that VAPB‐PTPIP51 knockdown aggravated the compromission of MAMs structure resulted from CIRI.

### 3.4 Knockdown of VAPB‐PTPIP51 aggravated oxidative stress in the brain after CIRI

**FIGURE 4:**
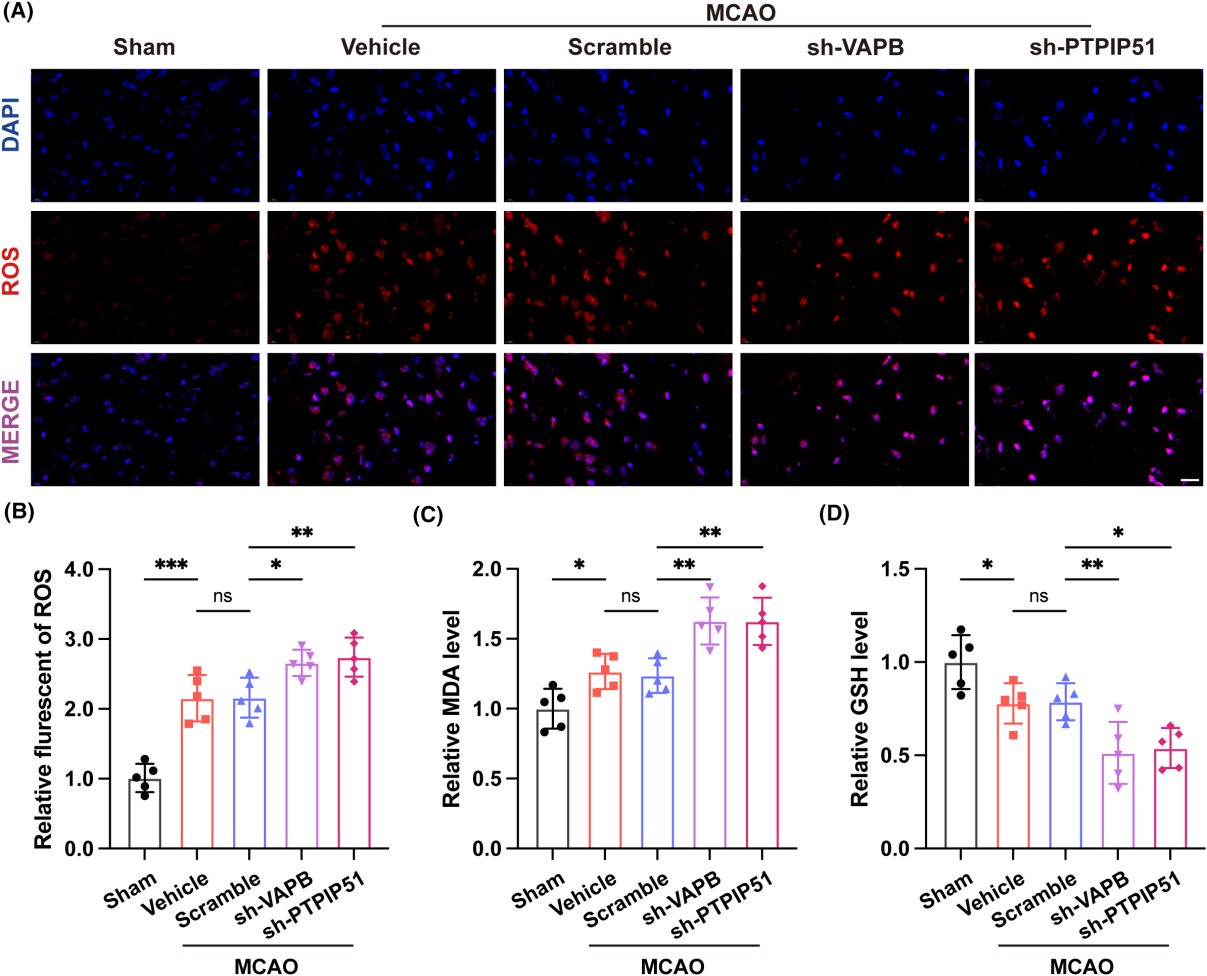
The effect of vesicle‐associated membrane protein‐associated protein B (VAPB)–PTPIP51 knockdown on oxidative stress after middle cerebral artery occlusion (MCAO). (A) Representative images of DHE staining showing the ROS level of mouse brain after VAPB or PTPIP51 knockdown. Scale bar = 20 μm, *n* = 5. (B–D) Relative levels of ROS, MDA and GSH. *n* = 5. All values represent mean ± SD, ns, nonsignificance, *p < 0.05, **p < 0.01, ***p < 0.001.

The destruction of MAMs results in oxidative stress, which is an important cause of brain tissue damage following CIRI.^38^ Therefore, we detected oxidative stress‐related indicators in the brain tissue, including ROS, MDA, and GSH. The results revealed that, compared with that in the sham operation group, the levels of ROS and MDA in the brain tissue increased, while GSH levels decreased after MCAO. In contrast to the vehicle group, the scramble group did not exhibit any significant differences in ROS, MDA, or GSH levels. However, compared to that in the scramble group, the knockdown of VAPB or PTPIP51 resulted in a further increase in ROS and MDA contents in the brain tissue, accompanied by lower GSH levels (Figure 4A–D). These results indicated that VAPB‐PTPIP51 knockdown aggravated oxidative stress in the brain tissue after CIRI.

### 3.5 Knockdown of VAPB‐PTPIP51 activated autophagy excessively in brain after CIRI

**FIGURE 5:**
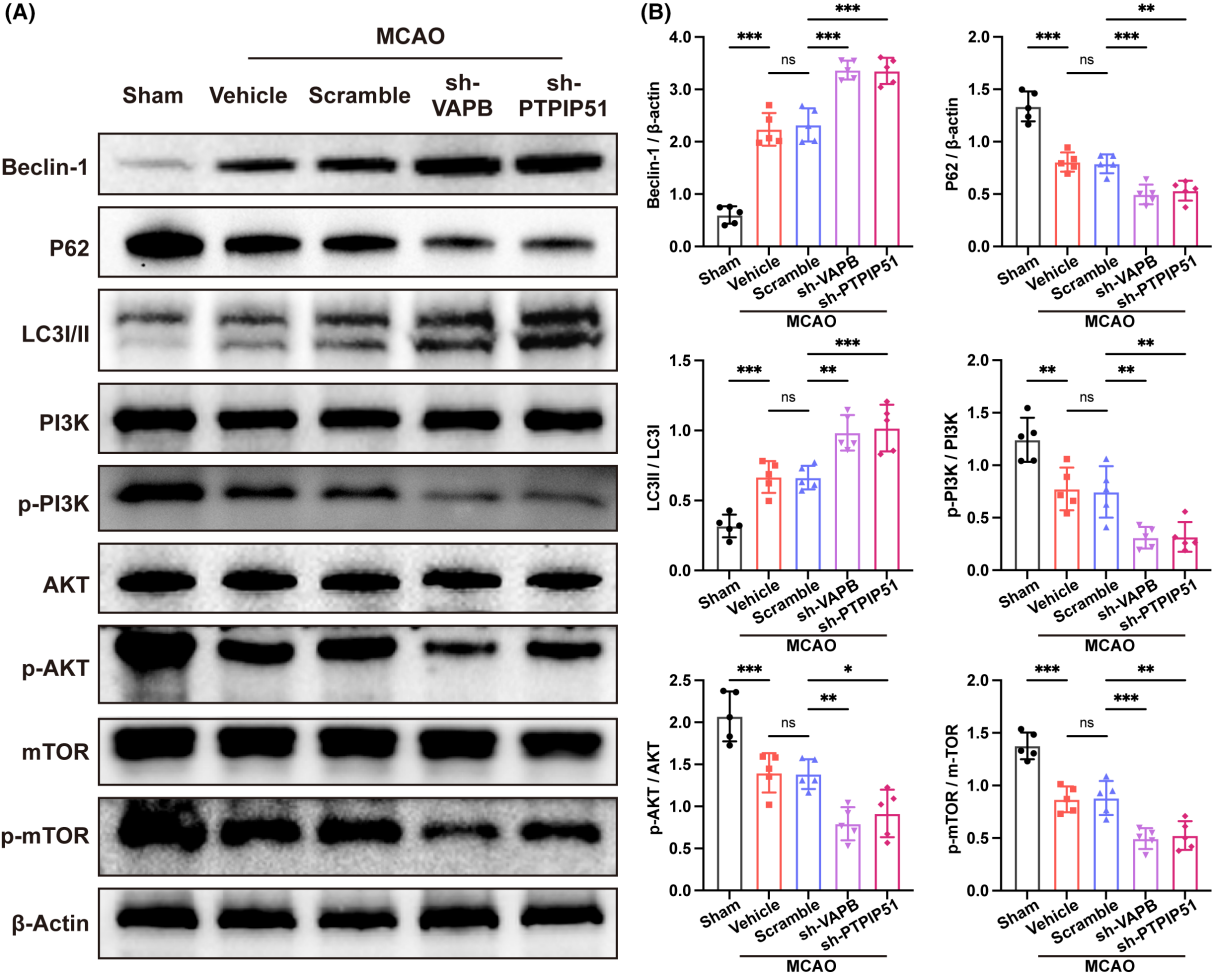
The effect of vesicle‐associated membrane protein‐associated protein B (VAPB)–PTPIP51 knockdown on autophagy and PI3K/AKT/mTOR signaling after middle cerebral artery occlusion (MCAO). (A) Representative Western blot bands of the protein expression of Beclin‐1, P62, LC3I/II, and PI3K/AKT/mTOR pathway in mice brains after MCAO following VAPB or PTPIP51 knockdown. *n* = 5. (B) Quantitative analysis of the expression of Beclin‐1, P62, LC3II/LC3I, p‐PI3K/PI3K, p‐AKT/AKT, and p‐mTOR/mTOR detected by Western blot. All values represent mean ± SD, ns, nonsignificance, *p < 0.05, **p < 0.01, ***p < 0.001.

MAMs are believed to regulate autophagy,^22^, ^23^ therefore, we hypothesized that the destruction of MAMs may cause autophagy disorders. In addition, the imbalance between the oxidative and antioxidant systems promotes the activation of autophagy, and excessive autophagy causes brain tissue damage after CIRI.^39^, ^40^ Compared to that in the sham group, the expression of Beclin‐1 was upregulated, the level of P62 was downregulated, and the rate of LC3II/LC3I was increased after CIRI. Notably, compared with that in the vehicle group, VAPB or PTPIP51 knockdown further upregulated Beclin‐1 and LC3II/LC3I and lowered P62 (Figure 5A,B). These results indicated that autophagy was activated after CIRI, and VAPB or PTPIP51 knockdown resulted in overactivated autophagy.

The PI3K/AKT/mTOR signaling pathway has been shown to mediate the autophagy.^41^ Western blot was performed to detect the change of PI3K/AKT/mTOR signaling after VAPB or PTPIP51 knockdown. The results showed that PI3K/AKT/mTOR signaling was inhibited after CIRI, as manifested by decreased rate of p‐PI3K/PI3K, p‐AKT/AKT, and p‐mTOR/mTOR. Furthermore, the levels of p‐PI3K/PI3K, p‐AKT/AKT, and p‐mTOR/mTOR were downregulated following VAPB or PTPIP51 knockdown after CIRI (Figure 5A,B).

### 3.6 Treatment with 740 Y‐P suppressed overactivated autophagy and mitigated aggravated CIRI following VAPB‐PTPIP51 knockdown

**FIGURE 6:**
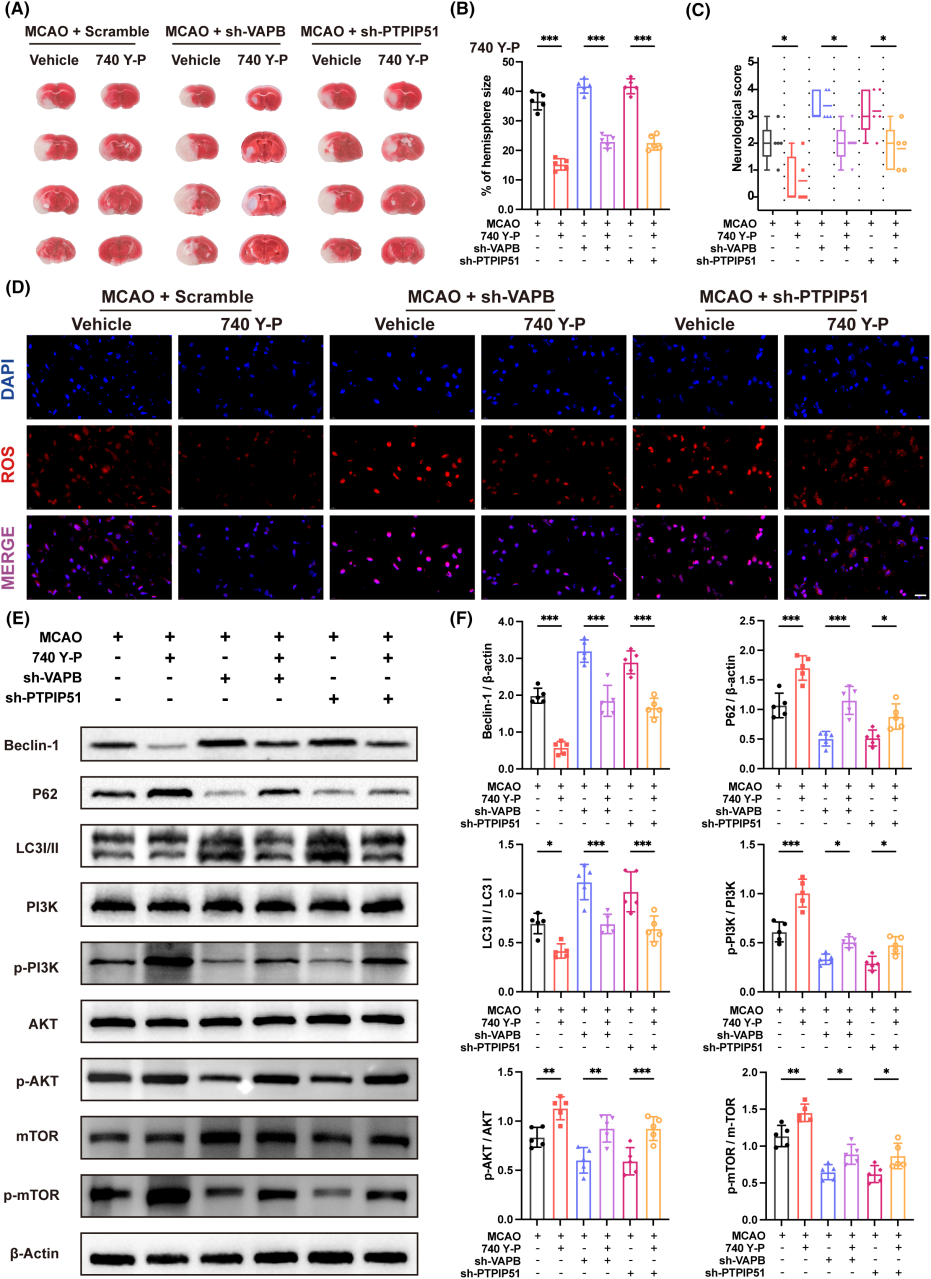
The effect of PI3K activator 740 Y‐P on vesicle‐associated membrane protein‐associated protein B (VAPB)–PTPIP51 knockdown mice after middle cerebral artery occlusion (MCAO). (A) Representative TTC staining of PI3K activator treated VAPB or PTPIP51 knockdown mice brain after MCAO. *n* = 5. (B) Quantitative analysis of the infarct size. *n* = 5. (C) Neurological deficits of VAPB or PTPIP51 knockdown mice treated with PI3K activator. *n* = 5. (D) ROS staining of VAPB or PTPIP51 knockdown mice brain with PI3K activator treatment. Scale bar = 20 μm, *n* = 5. (E) Western blot bands of the protein expression of autophagy flow and PI3K/AKT/mTOR pathway in 740 Y‐P treated mouse brains after VAPB or PTPIP51 knockdown. *n* = 5. (F) Quantitative analysis of the expression of Beclin‐1, P62, and the rate of LC3II/LC3I, p‐PI3K/PI3K, p‐AKT/AKT, and p‐mTOR/mTOR detected by Western blot. All values represent mean ± SD, *p < 0.05, **p < 0.01, ***p < 0.001.

To further validate whether VAPB or PTPIP51 aggravates CIRI by regulating autophagy through the PI3K/AKT/mTOR signaling pathway, we treated mice with the PI3K activator. Consistent with these results, compared with the vehicle mice, the PI3K activator reduced the infarct volume and alleviated the neurological deficits in scramble‐transfected mice after CIRI. Moreover, the activation of PI3K pathway showed a protective effect in VAPB or PTPIP51 knockdown mice after CIRI. This manifested as a partial reduction in the cerebral infarct volume and neurological deficit score (Figure 6A–C). Levels of ROS, MDA, and GSH were determined. The results revealed that PI3K activator treatment partly decreased the levels of ROS and MDA and increased the level of GSH, indicating that PI3K activation could alleviate VAPB or PTPIP51 knockdown‐induced excessive oxidative stress after CIRI (Figure 6D, Figure S1). In addition, we detected the expression of proteins related to the PI3K/AKT/mTOR signaling pathway and autophagy. Our results showed that PI3K activator treatment upregulated the p‐PI3K, p‐AKT, and p‐mTOR in VAPB or PTPIP51 knockdown mice with CIRI. Furthermore, the activation of PI3K pathway downregulated the Beclin‐1 and LC3II/LC3I, and upregulated the P62, compared to that in the vehicle group in VAPB or PTPIP51 knockdown mice with CIRI (Figure 6E,F). These results indicate that the PI3K/AKT/mTOR signaling pathway mediates VAPB or PTPIP51 deficits‐induced brain injury in IS mice.

## 4 DISCUSSION

IS presents with high incidence, disability, mortality, and recurrence rates.^42^ The intricate pathological mechanisms of IS persist as a puzzle, thus in‐depth exploration is urgently required to identify innovative therapeutic targets. In this study, we found that MAMs are damaged after IS, and the damage to MAMs affects the prognosis of mice by activating autophagy through the PI3K/AKT/mTOR pathway.

MAMs serve as a critical bridge for communication between mitochondria and the ER, playing an essential role in lipid synthesis, Ca^2+^ homeostasis regulation, functional coordination, and the death signaling between these organelles.^43^ It has been implicated that the damage to MAM structures is associated with various diseases, including amyotrophic lateral sclerosis and frontotemporal dementia (ALS/FTD), Alzheimer's, and Parkinson's.^44^, ^45^, ^46^ For example, VAPB and PTPIP51 are key components in the MAMs structure and are linked to synaptic signaling.^25^ Research utilizing animal models of ALS/FTD has shown that the disruption of VAPB‐PTPIP51 tethers is an early event that precedes disease onset.^32^ Additionally, the overexpression of VAPB or PTPIP51 has been found to enhance ER–mitochondria signaling and promote synaptic signal transmission, suggesting the potential of targeting MAMs for therapeutic interventions in neurodegenerative diseases like ALS/FTD.^46^ Our study delved into the specific role of MAMs in CIRI. A significant decrease in VAPB and PTPIP51 expression and structural damage of MAMs were observed in mice brains following CIRI, implying a role for MAMs in CIRI‐induced neuronal damage. To verify this hypothesis, we employed the LV injection to knockdown the expression of VAPB or PTPIP51 in mouse brains, thereby observed a worsened prognosis. Compared with the scramble group, the knockdown of VAPB or PTPIP51 resulted in an increased brain infarct volume and a worsen neurological deficit, indicating a correlation between MAMs and the outcomes of CIRI in mice. However, the study acknowledges a limitation in that it did not conduct complementary experiments involving the overexpression of VAPB and PTPIP51, which could have provided more robust support for the findings.

Autophagy, a rigorously regulated intracellular degradation and recycling system, is essential for the removal of damaged organelles.^47^ Previous research has shown that MAMs may serve as formation sites for autophagosomes, contributing to the initiation of autophagy.^48^ Additionally, MAMs are crucial in the transfer of Ca^2+^ from the ER to mitochondria.^15^, ^49^ The disruption of Ca^2+^ mediated by IP3R may trigger the activation of autophagy.^22^ Oxidative stress, driven predominantly by mitochondrial ROS, is a critical factor in pathological damage following IS.^10^, ^50^ MAMs are involved in this process as well, with structural and functional changes in these membranes influencing mitochondrial ROS production.^51^ In this study, we found that ROS increased in brain tissue after CIRI, accompanied by the activation of autophagy, which is consistent with existing reports.^10^, ^50^ However, more importantly, we found that knockdown of VAPB or PTPIP51 further exacerbated oxidative stress and autophagy in the brain tissue of CIRI mice. In the study, it was observed that compared to the scramble group, knockdown of VAPB or PTPIP51 resulted in increased levels of ROS and MDA, a decline in GSH, and alterations in autophagy‐related proteins. Specifically, there was a decrease in P62, alongside an increase in Beclin‐1 and the ratio of LC3II to LC3I. This result indicates that after CIRI, MAMs damage can cause excessive oxidative stress and autophagy, thereby aggravating brain tissue damage. However, the specific mechanism of this process is not completely clear.

Studies have demonstrated that the PI3K/AKT/mTOR signaling pathway is inhibited after CIRI, correlating with excessively increased oxidative stress and autophagy.^52^, ^53^, ^54^ However, it is unclear whether impaired MAMs after CIRI affect oxidative stress and autophagy through PI3K/AKT/mTOR. The modulation of the PI3K/AKT/mTOR pathway may be influenced by factors such as Ca^2+^ transfer, ER stress, and mitochondrial function, all of which are closely associated with MAMs, highlighting the potential role of MAMs.^55^, ^56^, ^57^ Therefore, we speculated that post‐CIRI MAMs might mediate oxidative stress and autophagy through PI3K/AKT/mTOR and verified it. The results show that the PI3K activator can partially reverse the aggravation of injury in CIRI mice induced by VAPB or PTPIP51 reduction, which is manifested by attenuating oxidative stress and autophagy, reducing cerebral infarction volume and improving neurological function. However, considering the human body's complexity suggests potential involvement of other signaling pathways in MAMs' regulatory effects on oxidative stress and autophagy, which requires further in‐depth research.

In summary, our findings pave the way for further comprehensive research, particularly focusing on the development of specific autophagy inhibitors and precise modulation of autophagy‐related genes, which could profoundly enhance the accuracy of analyzing the involvement of autophagy in MAMs‐induced CIRI. This study proposes a prospective direction for future research, offering novel insights into the development of therapeutic strategies for CIRI by targeting MAMs.

## AUTHOR CONTRIBUTIONS

Xiaoxing Xiong, Lijuan Gu, and Zhihong Jian designed this work and revised the manuscript. Mingyang Li and Yonggang Zhang wrote the manuscript and performed the experiments. Guixiang Yu prepared the figures and submitted the article. Hua Zhu and Shi Feng collected the literature. Mingyang Li and Yonggang Zhang performed bioinformatics analysis, Xiaoxing Xiong and Zhihong Jian revised the manuscript. All authors contributed to the manuscript revision and read and approved the submitted version.

## FUNDING INFORMATION

This work was funded by grants from the National Natural Science Foundation of China (grant no. 82271370 to Lijuan Gu and 82371346 to Xiaoxing Xiong).

## CONFLICT OF INTEREST STATEMENT

The authors have no conflicts of interest regarding the publication of this paper to declare.

## Supporting information


Appendix S1.

Figure S1.

## Data Availability

The data presented in the study are included in the article/Supplementary Material, and further inquiries can be directed to the corresponding authors.
