# Supplementary figures and images for: Mitochondria‐associated endoplasmic reticulum membranes tethering protein VAPB‐PTPIP51 protects against ischemic stroke through inhibiting the activation of autophagy
Source: CNS Neurosci Ther. 2024 Apr 7;30(4):e14707. doi: 10.1111/cns.14707 (PMC10999572; doi:10.1111/cns.14707)

Figure 1A

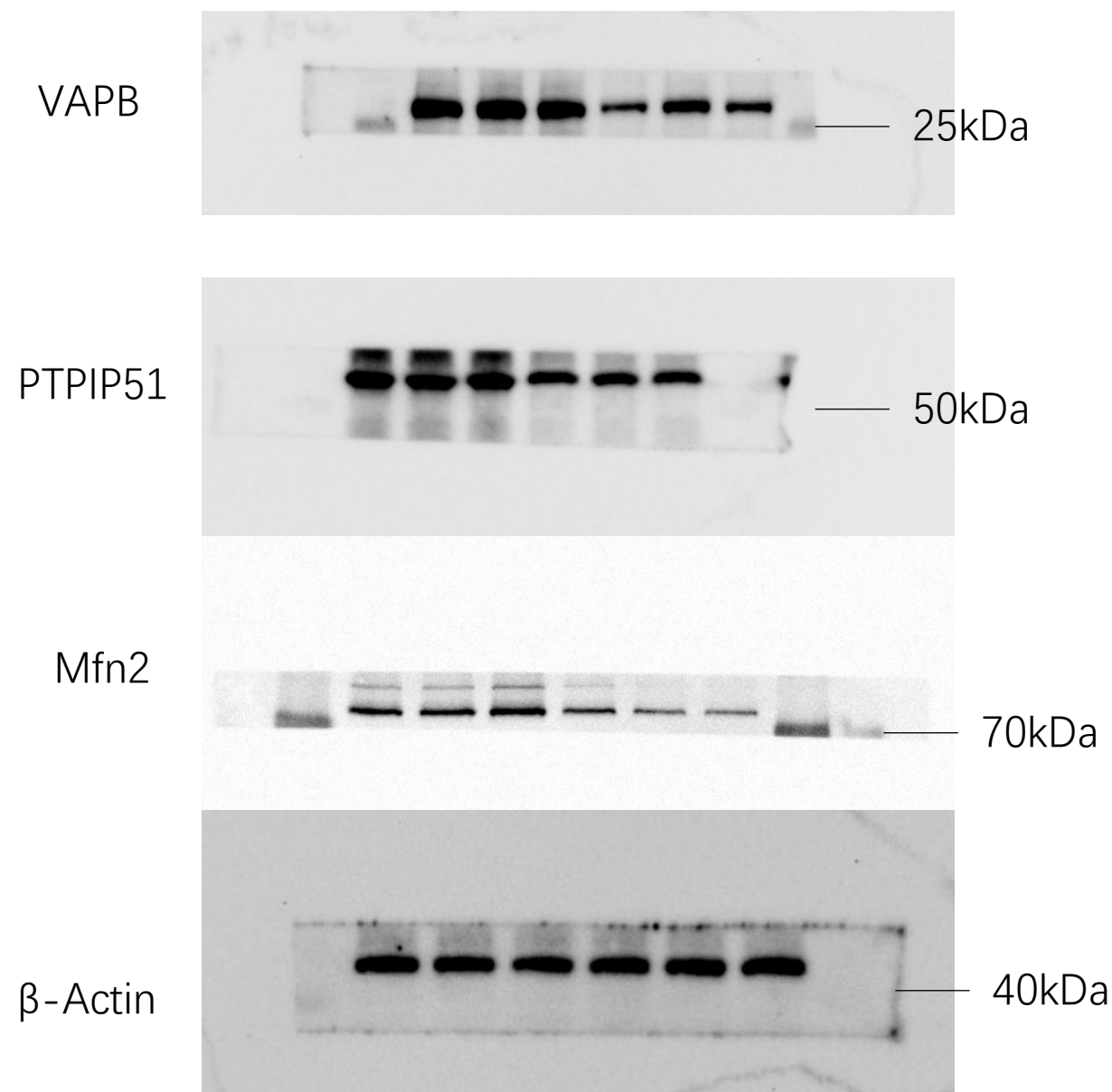

Figure 3A

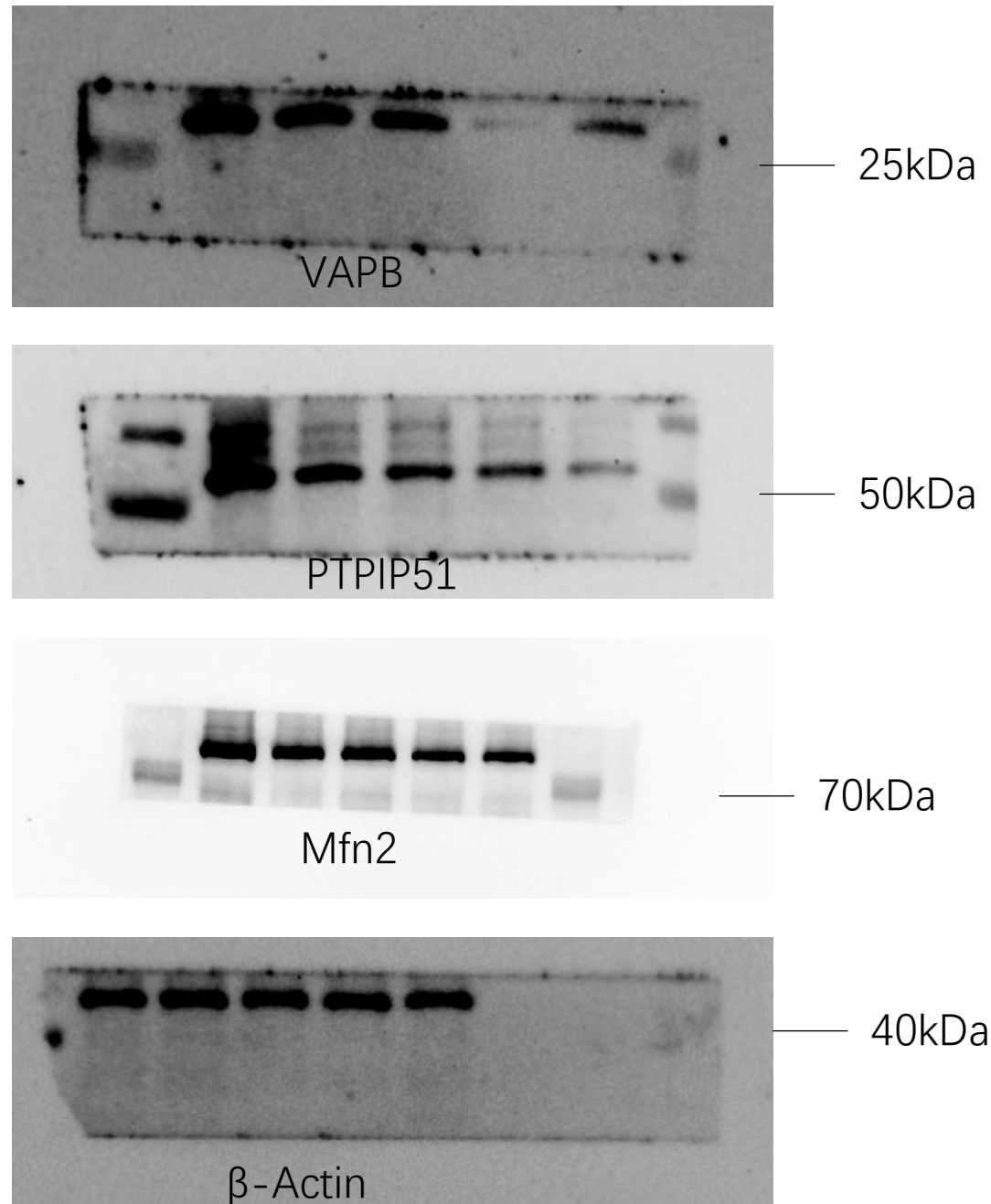

Figure 5A

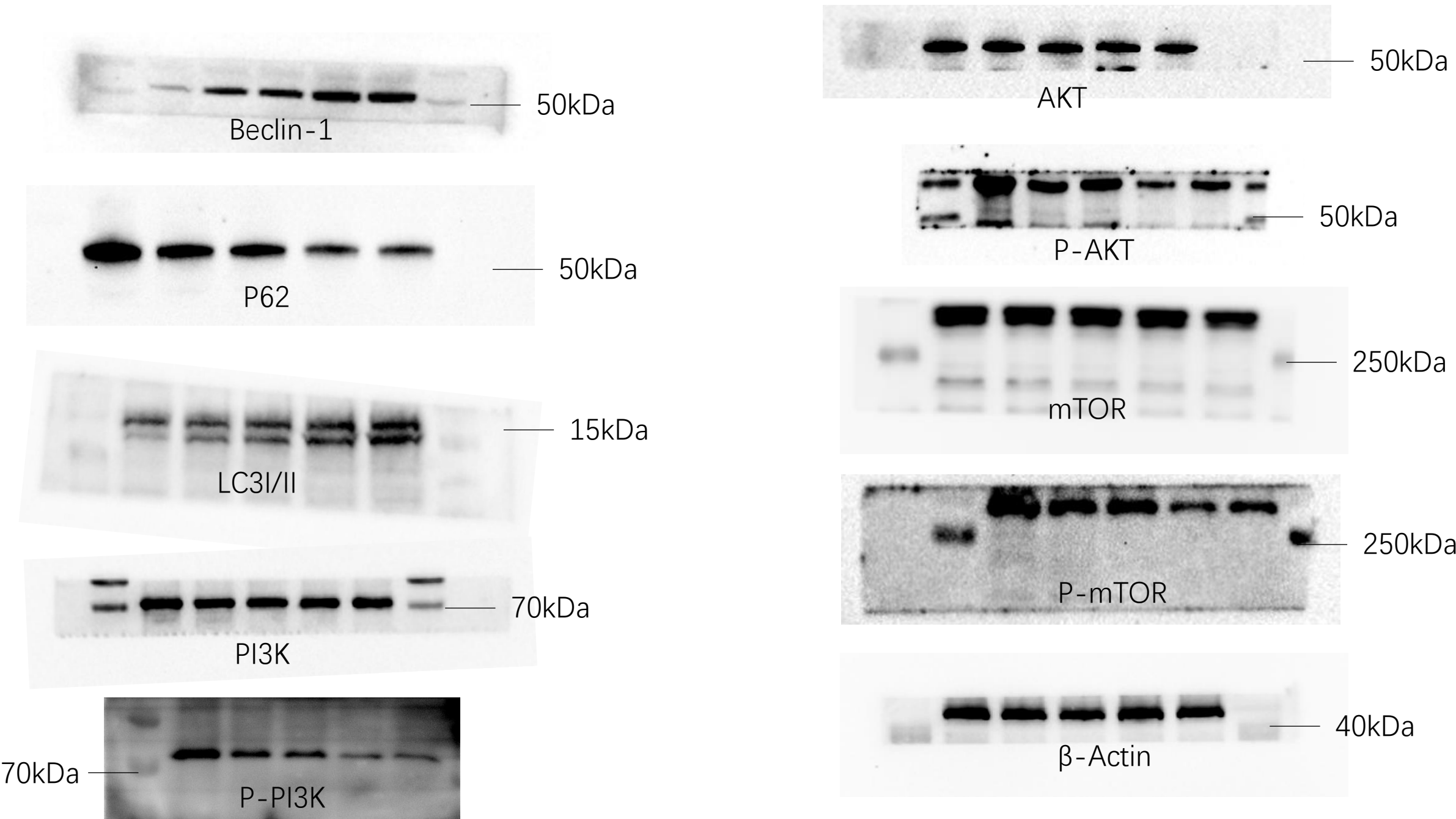

Figure 6A

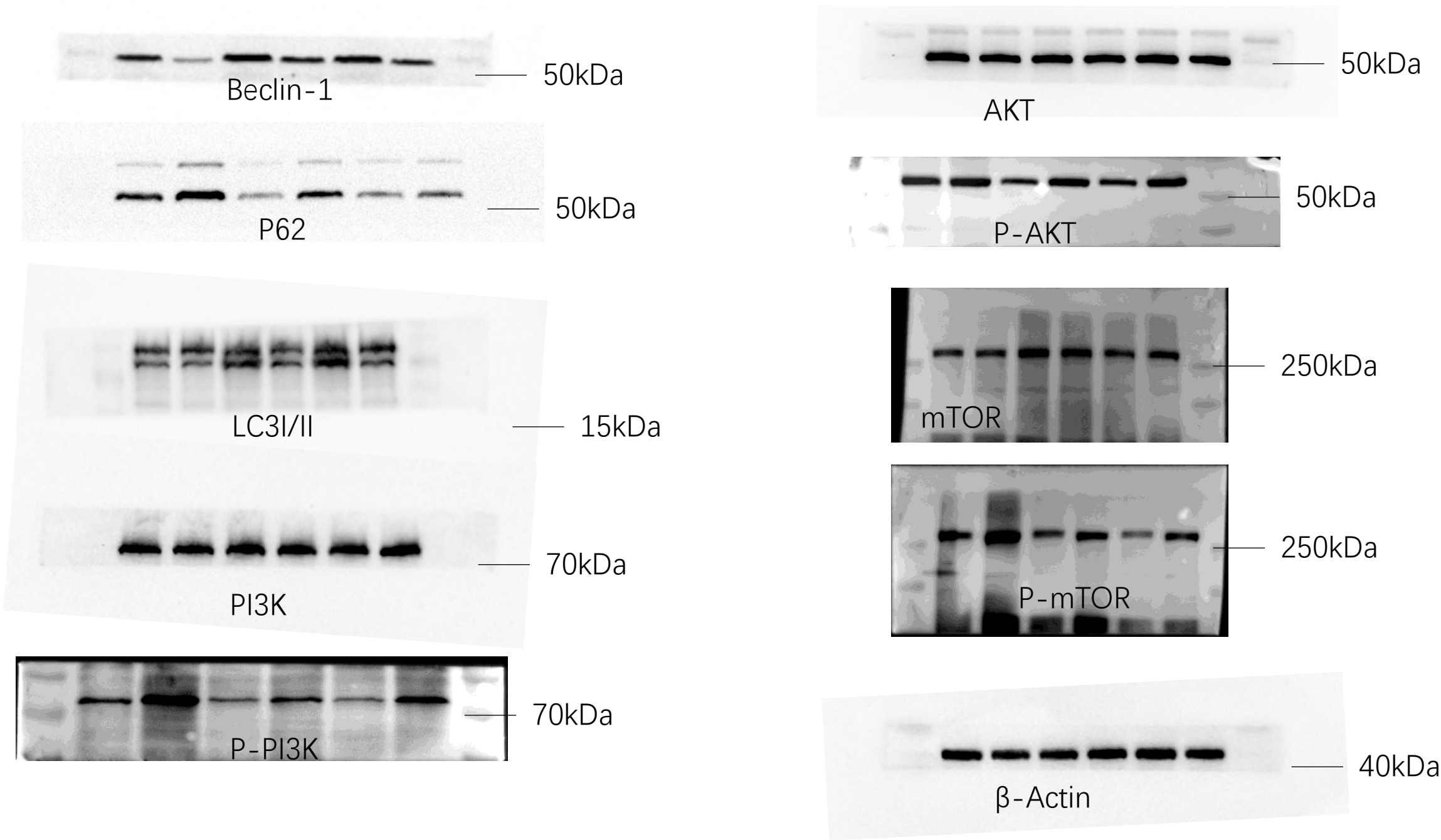

Supplement: Supplementary file 1 — Appendix S1. [file CNS-30-e14707-s002.pdf]
