# Supplementary material for: Mitochondria‐associated endoplasmic reticulum membranes tethering protein VAPB‐PTPIP51 protects against ischemic stroke through inhibiting the activation of autophagy
Source: CNS Neurosci Ther. 2024 Apr 7;30(4):e14707. doi: 10.1111/cns.14707 (PMC10999572; doi:10.1111/cns.14707)
Supplement: Supplementary file 2 — Figure S1. [file CNS-30-e14707-s001.docx]

**Supplementary material**


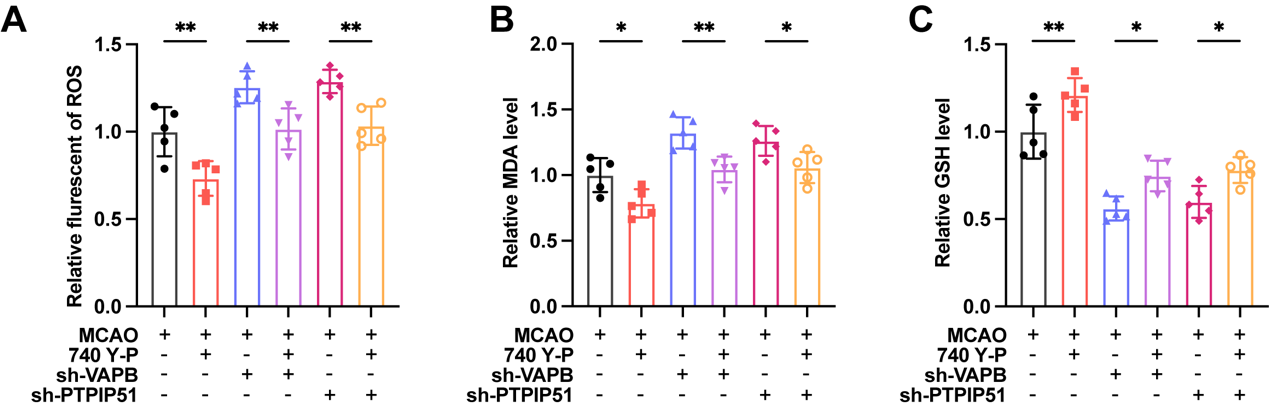


Figure S1. The effect of 740 Y-P on oxidative stress in VAPB-PTPIP51 knockdown mice after MCAO.

A-C. Relative levels of ROS, MDA and GSH in 740 Y-P treated VAPB or PTPIP51 knockdown mice brain after MCAO. n = 5. All values represent mean ± SD, *P < 0.05, **P < 0.01.
